# Comparing Real-Time PCR and Amplicon-Based NGS for Routine *EGFR* Liquid Biopsy Profiling

**DOI:** 10.3390/diagnostics16152392

**Published:** 2026-07-30

**Authors:** Andrea Boscolo Bragadin, Valeria Tosello, Stefano Longo, Alessia Padovan, Sara Baldissin, Paola Del Bianco, Elisa Masetto, Francesco Callegarin, Laura Bonanno, Giulia Pasello, Valentina Guarneri, Stefano Indraccolo

**Affiliations:** 1 Basic and Translational Oncology Unit, Veneto Institute of Oncology IOV-IRCCS, 35128 Padua, Italy; 2 Department of Surgery, Oncology and Gastroenterology, University of Padua, 35124 Padua, Italy; 3 Clinical Research Unit, Veneto Institute of Oncology IOV-IRCCS, 35128 Padua, Italy; 4 Medical Oncology 2, Veneto Institute of Oncology IOV-IRCCS, 35128 Padua, Italy

**Keywords:** lung cancer, NSCLC, liquid biopsy, NGS, real-time PCR, EGFR, cfDNA, ctDNA

## Abstract

**Background:** Sensitive detection of *EGFR* mutations in liquid biopsies of advanced non-small-cell lung cancer (aNSCLC) is vital for guiding targeted treatments. Real-time PCR offers a quick turnaround time but relatively low sensitivity while Next-Generation Sequencing (NGS) offers broader *EGFR* coverage, co-mutation evaluation and high sensitivity. **Methods:** This study evaluated the amplicon-based NGS Plasma-SeqSensei™ Solid Cancer In Vitro Diagnostics (IVD) Kit (Sysmex) against the real-time PCR-based cobas^®^ EGFR Mutation Test v2 (Roche), the current routine standard at the Veneto Institute of Oncology IOV-IRCCS. We enrolled 130 patients with aNSCLC in the RARE Study between April 2022 and August 2025 who were referred to our institute. Liquid biopsies were taken at diagnosis or at progression and analyzed using two methods with the primary objective of assessing diagnostic concordance for *EGFR* profiling. Sysmex NGS libraries were sequenced on a NextSeq 550 sequencer (Illumina), with the NextSeq 500/550 Mid Output Kit v2.5 (150 Cycles) in single-end mode. **Results:** Among 129 evaluable samples, the NGS assay demonstrated a marginally higher *EGFR* mutation detection rate, identifying mutations in 31/129 cases (24.0%, 95% CI: 17–33), versus 28/129 (21.7%, 95% CI: 15–30) by cobas, specifically for variants covered by both assays. Overall concordance was almost perfect (Cohen’s Kappa = 0.89, 95% CI: 0.80–0.98), confirming the high reliability of both methods. Furthermore, we identified a cfDNA input threshold of at least 20 ng as critical for ensuring optimal assay sensitivity and reliable mutation detection (Odds Ratio = 4.41 for inputs ≥ 20 ng, *p* = 0.006). **Conclusions:** Ultimately, the Sysmex NGS proved to be a robust and highly sensitive assay. It delivers performance comparable to that of standard RT-PCR while providing the clinical advantage of concurrently detecting a broader spectrum of *EGFR* variants and actionable mutations in other genes.

## 1. Introduction

Lung cancer is the leading cause of cancer-related death worldwide, with non-small-cell lung cancer (NSCLC) accounting for 85% of all cases [1]. The detection of driver oncogenic alterations allows patients to access targeted therapies, which have revolutionized treatment of lung cancer over the last decade [2,3,4,5,6]. *Epidermal Growth factor receptor* (*EGFR*) mutations represent one of the most frequent somatic alterations, with a prevalence that varies significantly across regions, ranging from 14.1% in Europe to 17.2% in the Middle East/North Africa, reaching 49.1% in Asia [7]. This pooled prevalence is significantly higher among females, non-smokers, and patients with adenocarcinoma [8]. The most common *EGFR*-sensitizing mutations are found in exon 21 (e.g., p.(L858R)) and exon 19 deletions, while exon 20 insertions and exon 18 mutations occur less frequently. These oncogenic mutations constitutively activate the tyrosine kinase domain of the EGFR protein, leading to the downstream activation of the MAPK cascade, the PIK3CA-AKT-mTOR and the PLCγ-PKC pathways. This promotes uncontrolled cell proliferation and survival, ultimately sustaining tumor growth. The therapeutic landscape for *EGFR*-mutated advanced NSCLC has rapidly evolved over the last decade, progressing from first- and second-generation tyrosine kinase inhibitors (TKIs) (e.g., erlotinib, gefitinib, and afatinib) [9,10,11] to the third-generation TKI osimertinib, designed to target the *EGFR* p.(T790M) resistance mutation in exon 20. Subsequently, osimertinib established a new first-line standard of care [3,12], although resistance eventually emerges through the *EGFR* p.(C797S) mutation and various off-target mechanisms [13,14]. More recently, treatment paradigms have further advanced with upfront combination strategies, such as osimertinib plus platinum-pemetrexed (FLAURA2 trial) [15] and amivantamab plus lazertinib (MARIPOSA trial) [16]. Furthermore, novel combination regimens with the addition of chemotherapy to osimertinib are being actively investigated at baseline in patients harboring *EGFR*/*TP53* co-mutations (TOP trial) and upon disease progression (COMPEL trial) [17,18]. In this highly dynamic scenario, liquid biopsy has become an indispensable tool for tracking tumor evolution and acquired resistance. Crucially, the APPLE trial has underscored the clinical utility of liquid biopsy for dynamic circulating tumor DNA (ctDNA) monitoring by proposing an early switch to osimertinib before radiological progression based solely on the liquid biopsy detection of the T790M mutation [19].

Other targetable genetic alterations in lung cancer, for which specific treatments are currently approved in clinical practice, include *BRAF* p.(V600E) and *KRAS* p.(G12C) mutations, and *ALK*, *ROS1*, *RET*, and *NTRK* fusions [20]. Conversely, therapies targeting *PIK3CA* mutations have historically failed as monotherapies in lung cancer due to limited clinical response [21,22], indicating that *PIK3CA* is likely not a primary driver gene in NSCLC. Nevertheless, *PIK3CA* co-occurrence in *EGFR*-mutant tumors is a recognized mechanism of TKI resistance and may guide combination therapy [23,24,25,26]. This highly heterogeneous genetic landscape requires sensitive and timely molecular characterization to accurately guide therapeutic decisions and monitor treatment responses.

While tissue biopsy remains the historical gold standard for the molecular profiling of NSCLC, its clinical utility is frequently limited by poor accessibility, procedural invasiveness, and the inability to fully capture the spatial and temporal heterogeneity of the tumor. Consequently, liquid biopsy—based on the analysis of circulating cell-free DNA (cfDNA) in plasma—has established its role as a highly reliable, non-invasive alternative [27,28,29]. In the advanced disease setting, a ‘liquid-first’ approach is increasingly endorsed by international guidelines, particularly when tissue is scarce, exhausted, or difficult to obtain, as it significantly accelerates the time to treatment initiation [30,31]. Furthermore, unlike static tissue biopsies, liquid biopsy enables longitudinal assessment to dynamically monitor the disease course and track clonal evolution [32]. This real-time monitoring is crucial for the early identification of acquired resistance mechanisms during targeted therapy, allowing oncologists to swiftly adapt combination strategies without subjecting patients to repeated, high-risk invasive procedures.

The accurate identification of *EGFR* mutations plays a central role in the treatment planning of NSCLC. In the advanced disease setting, various liquid biopsy-based diagnostic tests are available to detect *EGFR* alterations. These tests primarily rely on technologies like real-time PCR and Next-Generation Sequencing (NGS), each offering unique advantages and limitations. The cobas^®^ EGFR Mutation Test v2 (Roche) is a real-time PCR-based method that represents the standard diagnostic assay in many laboratories due to its rapid turnaround time and its FDA regulatory approval [33]. However, targeted tests of this type are inherently limited to detecting specific, pre-defined alterations and often exhibit a relatively low sensitivity. Since circulating tumor DNA (ctDNA) typically constitutes a minute fraction, often less than 1%, of the total cfDNA, this limitation can potentially lead to false-negative results.

NGS-based tests are gaining prominence in liquid biopsy because they provide a more comprehensive molecular profile of the tumor [25]. NGS allows the simultaneous detection of the ESMO Scale for Clinical Actionability of Molecular Targets (ESCAT)-actionable genes [20,34], rare *EGFR* variants, novel mutations, including resistance mutations, and other potentially co-occurring actionable genes (such as *BRAF*, *KRAS* and *PIK3CA*) that may have prognostic and predictive value [23,26,32,35,36,37,38]. NGS often provides increased analytical sensitivity compared to real-time PCR [39]. On the downside, NGS platforms lack universal standardization, incur higher costs, and require more hands-on time and specialized bioinformatics personnel.

In this study, we evaluated the performance of the highly sensitive, amplicon-based NGS Plasma-SeqSensei™ Solid Cancer IVD Kit (Sysmex) in comparison to the routine real-time-based cobas^®^ EGFR Mutation Test v2 for *EGFR* profiling in liquid biopsy. This NGS test relies on Safe-SeqS technology [40] and allows the detection of genetic alterations across five key genes including *EGFR*, *BRAF*, *KRAS*, *PIK3CA* and *NRAS* (covering 17 amplicons) with a certified sensitivity down to 0.07% variant allele fraction (VAF), or 7 mutant molecules within a background of 10,000 wild-type copies. Additionally, it features a relatively rapid wet-lab protocol and holds a European IVD certification that covers the entire workflow, down to the software and the final clinical report [41]. Here, we retrospectively and prospectively enrolled 130 aNSCLC patients undergoing routine plasma testing with the primary objective of assessing the analytical concordance between the two methods for detecting *EGFR* mutations.

## 2. Materials and Methods

### 2.1. Study Design and Patient Enrollment

Patients with histologically confirmed advanced NSCLC undergoing routine diagnostic *EGFR* testing at the Veneto Institute of Oncology IOV-IRCCS were enrolled in the RARE Study between April 2022 and August 2025. This was a single-center, blinded, cross-sectional, retrospective–prospective Post-Market Performance Follow-up (PMPF) study. The primary objective was to assess the diagnostic concordance for plasma *EGFR* profiling between the assay currently used in routine practice, the real-time PCR cobas^®^ EGFR Mutation Test v2 (Roche Diagnostics, Basel, Switzerland), and the NGS-based Plasma-SeqSensei™ Solid Cancer IVD Kit (Sysmex Inostics GmbH, Hamburg, Germany). To ensure an unbiased evaluation, all tests were performed by two independent operators, each strictly blinded to the results of the alternate assay. The inclusion criteria for the study were as follows: male or female patients aged 18 years or older; patients with advanced or metastatic non-small-cell lung cancer (NSCLC) at diagnosis or disease progression who had previously undergone or were scheduled to undergo liquid biopsy testing with the cobas^®^ EGFR Mutation Test v2, yielding a valid result (either positive or negative); and the availability of at least 6 mL of plasma. Patients were excluded from the study if there was insufficient plasma volume available to perform both tests or if the cobas^®^ assay yielded an invalid result. The local ethics committee, CET-ANV (Comitato Etico Area Nord Est Veneto), approved the study design and the informed consent protocol (approval number: 2024-35; 5 June 2024). Written informed consent was obtained from all patients prior to study entry. The study was conducted in full accordance with the precepts of the Declaration of Helsinki.

### 2.2. Plasma Sample Collection

Peripheral blood samples were collected from each patient as part of the routine *EGFR* diagnostic workup at the Veneto Institute of Oncology IOV-IRCCS. Briefly, blood was drawn into two Cell-Free DNA BCT^®^ tubes (Streck, La Vista, NE, USA) and centrifuged at 2000× *g* for 10 min. The resulting supernatant was subsequently centrifuged at 20,000× *g* for 10 min to pellet cellular debris. The final processed plasma was stored at −80 °C until needed for cobas and NGS testing. No additional blood draws were required for the NGS analyses; all tests were performed using residual plasma from the routine *EGFR* diagnostic testing.

### 2.3. cfDNA Extraction and Quantification

For the cobas^®^ EGFR Mutation Test v2, cfDNA was isolated from 2 mL of plasma using the cobas^®^ cfDNA Sample Preparation Kit, following the manufacturer’s instructions. The extraction kit is provided within the real-time PCR kit and is required by the IVD protocol. Quantification was performed using the Qubit™ dsDNA HS Assay Kit on a Qubit 4.0 fluorometer (Thermo Fisher Scientific, Waltham, MA, USA), utilizing a 2 μL input per sample.

For NGS testing, cfDNA was extracted from 2 to 5 mL of plasma using the semi-automated Maxwell^®^ RSC ccfDNA Large Volume (LV) kit on a Maxwell^®^ CSC Instrument (Promega, Madison, WI, USA), in accordance with the manufacturer’s protocol. Elution was performed using the NGS buffer provided in the extraction kit. The elution volume was specifically adjusted to 155 μL to accommodate the Sysmex NGS protocol, which requires a minimum input volume of 130 μL (accounting for expected buffer evaporation during the procedure). The extracted cfDNA was quantified via the Qubit™ dsDNA HS Assay Kit, using an input of 5 μL per sample, as specified in the Sysmex manual. Although the manufacturer’s protocol for the Sysmex NGS assay recommends the QIAamp Circulating Nucleic Acid Kit (QIAGEN, Hilden, Germany) for cfDNA extraction, we opted to use the Promega Maxwell^®^ RSC ccfDNA LV kit. This deviation from the standard IVD workflow was implemented to accommodate the high-throughput demands of a routine diagnostic setting. The automated Promega system significantly reduces manual hands-on time, minimizes the risk of cross-contamination, and standardizes extraction efficiency, making it more suitable for daily clinical practice.

### 2.4. cobas^®^ EGFR Mutation Test v2

The cobas^®^ EGFR Mutation Test v2 was performed as outlined in the manufacturer’s manual on a cobas^®^ z 480 analyzer (Roche Diagnostics, Basel, Switzerland). Briefly, this real-time PCR assay utilizes three Master Mix (MMX) solutions and magnesium acetate (MGAC). Each MMX was mixed with MGAC (20 μL + 5 μL) and distributed onto a 96-well plate, after which 25 μL of the sample is added to each well. The plate is then loaded into the cobas^®^ z 480 analyzer. Results were analyzed on the cobas 4800 software version 2.2.0.1509. To determine run validity, one positive control and two no-template controls (a water extraction control and an elution buffer control) were included and analyzed in every run. The software reports the detected mutations along with their corresponding Semi-Quantitative Index (ISQ). The ISQ is a proprietary metric, derived from the real-time PCR cycle threshold, that estimates the proportion of mutant DNA relative to the wild-type background.

### 2.5. NGS Libraries Preparation and Sequencing

Sequencing libraries were prepared using the Plasma-SeqSensei™ Solid Cancer IVD Kit, according to the manufacturer’s instructions. Briefly, 16 cfDNA samples were processed per library preparation session, alongside a positive control and a no-template control provided within the kit. The protocol requires a minimum of 5.7 ng up to 95 ng of cfDNA in input for each sample, measured by Qubit dsDNA HS. Where available, 43 ng of cfDNA was used as input, as recommended by the protocol. If a sample exhibited a higher concentration, it was diluted to achieve exactly 43 ng in 116 μL. Each cfDNA sample was divided across 5 wells (quintuplicate) on a 96-well plate and amplified via multiplex PCR targeting the specific panel regions. This amplification step simultaneously incorporates unique molecular barcode sequences, which allows significant reduction of background noise. A spike-in of artificial DNA at a known concentration (Quantispike) was added to the multiplex PCR mix to enable the absolute quantification of input cfDNA (expressed in Genome Equivalents, GE) and the precise calculation of mutant molecules of variants by the Plasma-SeqSensei™ IVD Software version 1.4.0 (Sysmex Inostics GmbH, Hamburg, Germany) after the sequencing. The amplified libraries were purified using AMPure XP Beads (Beckman Coulter, Brea, CA, USA), ligated to Illumina adapters, indexed, pooled together, and subsequently purified to obtain the final pooled library. Final library quantity and quality were assessed using the D1000 ScreenTape Assay on a TapeStation System (Agilent Technologies, Santa Clara, CA, USA). Following Sysmex guidelines, the libraries were initially diluted to 2 nM, and subsequently diluted further to 0.9–1 pM in accordance with the Illumina Denature and Dilute Libraries Guide. Sequencing was performed using the NextSeq 500/550 Mid Output Kit v2.5 (150 Cycles) in single-end mode on a NextSeq 550 sequencer (Illumina, San Diego, CA, USA). The alignment and variant calling of the final FASTQ files generated by sequencing were performed utilizing the Plasma-SeqSensei™ IVD Software (version 1.4.0). The software generates a comprehensive PDF report for each sample, detailing sequencing metrics, calculated input genomic equivalents (GE), identified mutations, variant allele frequencies (VAFs), and the absolute number of mutant molecules per variant. Run validity is automatically assessed by the software, requiring four quality control parameters, the positive control, no-template control, sequencing depth, and sample quantification, to fall within predefined ranges. Additionally, the manufacturer-validated detection threshold is strictly set at a VAF of 0.07% or 7 mutant molecules for all targets; variants falling below these limits are automatically filtered out and not reported.

### 2.6. Statistical Analyses

Sample size determination was based on the hypothesis that the NGS Plasma-SeqSensei™ Solid Cancer IVD Kit yields a higher detection rate of *EGFR* mutations compared to the routine cobas^®^ EGFR Mutation Test v2. Assuming a positivity rate of 20% for the routine cobas test and 25% for the NGS assay (reflecting a 5% difference in marginal frequencies), with an expected 90% correlation between paired observations, a sample size of 130 pairs was calculated. This sample size provides 84% power to detect a significant difference at a two-sided significance level of 0.05. The primary endpoint of the study was the relative frequency of *EGFR*-mutated samples, calculated as the proportion of positive samples out of the total samples tested by each method. The secondary endpoint was the assessment of concordance between the two analytical methods, evaluated using Cohen’s Kappa statistic. Continuous variables were described as median (interquartile range); categorical variables were described as absolute and percentage frequencies. Correlations were calculated with the Spearman’s index. Linear and logistic regression models were used to study the associations between the VAF and ISQ and between gene detection and cfDNA input (ng) calculated from Genome Equivalents. All statistical analyses were performed using R version 4.5.1 (R Foundation for Statistical Computing, Vienna, Austria). Cohen’s kappa coefficient was calculated using the psych package 2.6.1 (William Revelle, Northwestern University, Evanston, IL, USA).

## 3. Results

### 3.1. Cohort Characteristics and NGS Quality Metrics

Between April 2022 and August 2025, a total of 130 patients with advanced NSCLC were enrolled in the RARE study, either at initial diagnosis (*N* = 88, 68.2%) or at the time of disease progression (*N* = 41, 31.8%) (Supplementary Table S1). Each patient underwent routine *EGFR* diagnostic testing at the Veneto Institute of Oncology IOV-IRCCS using the cobas^®^ EGFR Mutation Test v2. Residual plasma from this routine diagnostic testing was subsequently used to extract cfDNA for NGS analysis with the Sysmex Plasma-SeqSensei™ Solid Cancer IVD kit. To obtain sufficient cfDNA for NGS analysis, we started with a median of 4.5 mL of plasma per patient. Following extraction, the median cfDNA yield was 12.5 ng/mL (IQR: 8.3, 29.5) (Table 1). The median cfDNA input utilized for NGS, as quantified by the Qubit dsDNA HS assay, was 34 ng. Following sequencing, the Plasma-SeqSensei™ IVD Software 1.4.0 performed absolute quantification of input cfDNA, expressed in genome equivalents (GE). The median GE input was 7203, which virtually mirrors the input quantity measured with Qubit (Table 1). Of the 130 patients processed for NGS, one sample failed due to insufficient input quantity (<5.7 ng) and was excluded from the downstream analysis, resulting in a final cohort of 129 valid cases.

### 3.2. Concordance Between cobas and Sysmex Assays

Among the 129 valid paired samples, concordant results were observed in 124 cases, yielding an overall agreement of 96.1%. This included 27 samples testing positive for *EGFR* mutations by both methods and 97 samples testing negative (Table 2). Within the positive concordant cases, we identified 10 mutations p.(L858R) in exon 21, 12 deletions in exon 19, 4 insertions in exon 20, and one exon 18 p.(G719A) mutation (Supplementary Table S1). Overall, the Sysmex NGS assay demonstrated a marginally higher *EGFR* mutation detection rate compared to the routine cobas test. Specifically, the Sysmex method identified *EGFR* mutations in 31 out of 129 samples (24.0%, 95% CI: 17–33; Table 2), whereas the cobas test detected mutations in 28 out of 129 samples (21.7%, 95% CI: 15–30; Table 2). The absolute difference in detection rates between the two methods was 2.3% (95% CI: −8.7–13.3; Table 2). A paired McNemar’s test with continuity correction revealed no statistically significant discordance bias between the two methods (*p* = 0.371). The overall concordance between the two assays was very high (Cohen’s Kappa = 0.89, 95% CI: 0.80–0.98; Table 2), highlighting that both tests are highly reliable for *EGFR* profiling.

### 3.3. Discordant and Partially Concordant Cases

Discordant results were observed in 5 patients (Table 3A). In patient #91, an *EGFR* exon 19 deletion was detected exclusively by the cobas test. In contrast, the Sysmex NGS assay uniquely identified an *EGFR* mutation in 4 patients that had been missed by cobas (Table 3A). Interestingly, manual inspection of the BAM sequencing files for patient #91 revealed trace evidence of the exon 19 deletion (23 out of 89,578 reads). However, this corresponded to a VAF of 0.000256%, which falls drastically below the Sysmex software’s predefined detection threshold of 0.07%, explaining why the variant was automatically filtered out. Although the superior detection capability of Sysmex in these 4 cases did not reach formal statistical significance due to the cohort size, the identification of 4 additional patients eligible for targeted TKI therapy is of high clinical relevance.

Among the concordant cases, we additionally observed two instances of partial correspondence, where the NGS assay successfully detected the primary mutation identified by cobas while concurrently revealing a secondary *EGFR* alteration (Table 3B). Specifically, in patient #23, cobas detected an exon 19 deletion but failed to identify a concurrent p.(T790M) resistance mutation. Upon disease progression on gefitinib, Sysmex detected the p.(T790M) mutation, allowing the patient to transition appropriately to osimertinib. Similarly, in patient #63, both methods detected the p.(L858R) mutation, but Sysmex uniquely identified an acquired p.(C797S) mutation (a known mechanism of resistance to osimertinib), an alteration which is not covered by the cobas assay design.

### 3.4. Quantitative Correlation: ISQ vs. VAF

**Figure 1:**
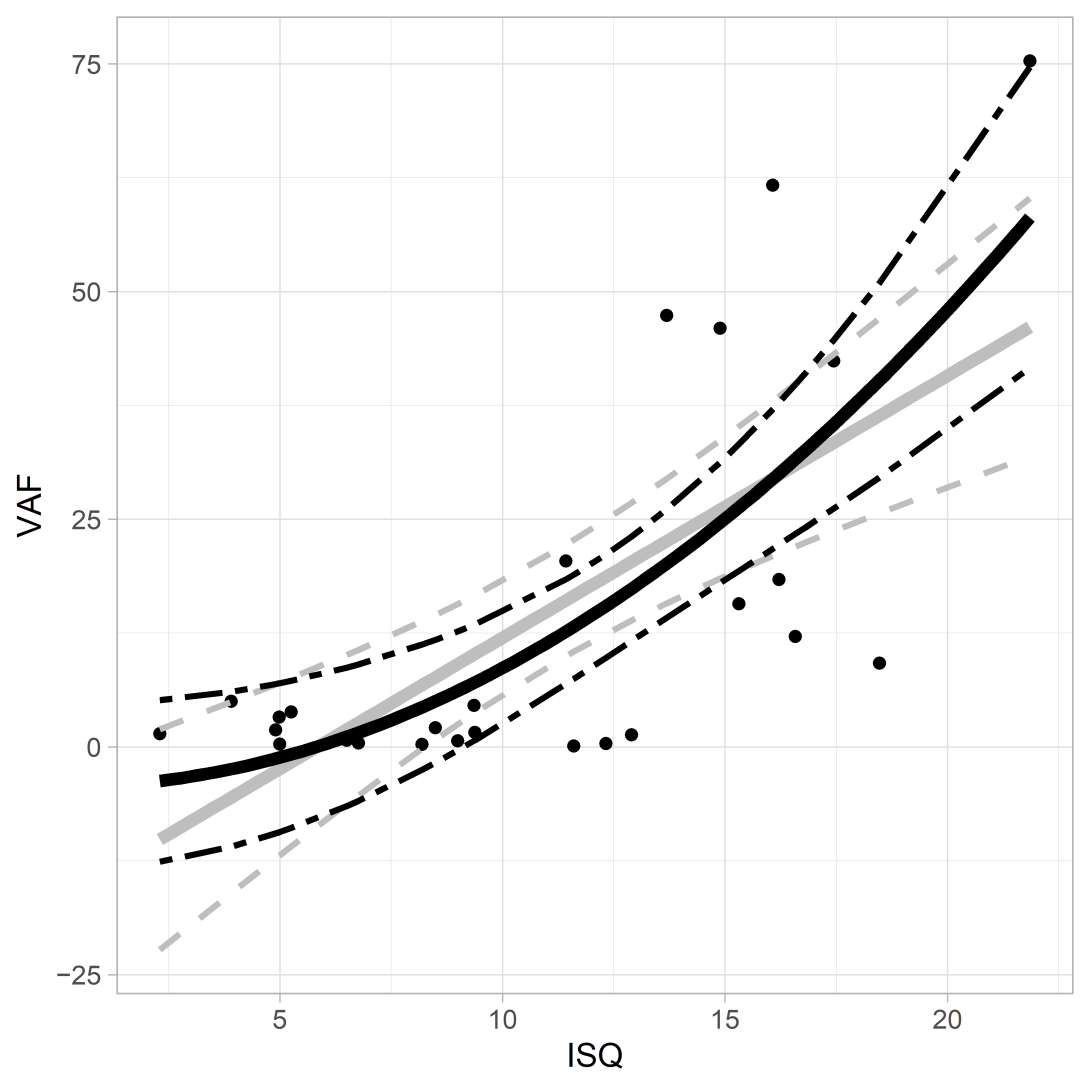
Scatter plot illustrating the relationship between the Semi-Quantitative Index (ISQ) derived from the real-time PCR assay and the Variant Allele Fraction (VAF) obtained via NGS. A significant positive correlation was observed (Spearman’s *ρ* = 0.61, *p* < 0.001). Each black dot represents an individual patient sample. The solid gray line represents the linear regression model, while the solid black line represents the quadratic regression model. Their respective 95% confidence intervals are indicated by the corresponding dotted lines.

Within the *EGFR*-mutated subgroup, the ISQ values from cobas and the VAF percentages from Sysmex presented medians of 10.40 (IQR: 6.6–15.7) and 1.70% (IQR: 0.5–11.2), respectively (Table 1). Focusing on the 27 samples with fully concordant *EGFR* mutations, we observed a statistically significant quantitative correlation between the cobas ISQ and the Sysmex VAF (Spearman’s *ρ* = 0.61, *p* < 0.001; Figure 1, Supplementary Table S2). This relationship was not perfectly linear but rather fits a quadratic model (β = 0.13, 95% CI: 0.08–0.18, *p* = 0.001, Figure 1, Supplementary Table S2). Notably, when the analysis was restricted exclusively to the subgroup of patients with an ISQ < 10, the correlation was lost (Supplementary Table S2).

### 3.5. Correlation Between Input cfDNA Quantity and Detection Rate of Mutations

We further investigated whether the quantity of input cfDNA utilized for NGS significantly influenced the mutation detection rate (any mutation). Univariate logistic regression revealed that for each 1-unit increase in nanograms in input (calculated from GE), the probability of successfully detecting any mutation increased by 8% (OR = 1.08, 95% CI: 1.03–1.12, Supplementary Table S3). Crucially, when the cfDNA input exceeded the threshold of 20 ng, the probability of detecting a mutation increased by 4.41 times (95% CI: 1.64–14.1, *p* = 0.006) compared to inputs below this cut-off (Supplementary Table S3). Finally, we evaluated whether the clinical status at the time of liquid biopsy collection (diagnosis vs. progression) affected the detection rate; however, no statistically significant difference was observed (*p* = 0.300) (Supplementary Table S3).

### 3.6. Co-Mutational Landscape Detected by Sysmex NGS

**Figure 2:**
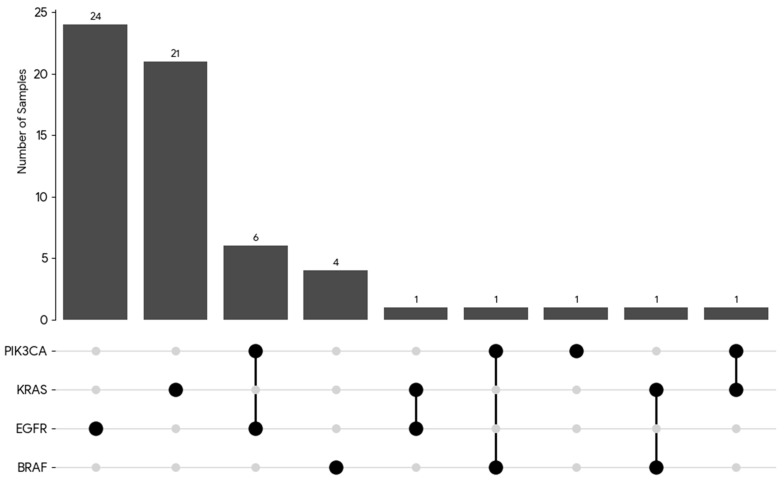
The UpSet plot illustrates the co-mutation landscape in patient plasma samples. Vertical bars on the top side represent the number of samples presenting either a unique single mutation or a specific combination of co-mutations, with the connecting dots (full black) on the bottom side indicating the genes involved in each subset, while the light gray dots represent the absence of mutations in the respective genes for that subset. All the mutations reported here have a VAF > 0.07%.

Beyond *EGFR*, the broader panel of the Sysmex NGS assay showed the advantage of capturing the genetic heterogeneity of the disease in both *EGFR*-mutated and non-*EGFR*-mutated patients (Figure 2).

Among the *EGFR*-mutated cohort, 24 patients exhibited an isolated *EGFR* mutation, 6 harbored concurrent *EGFR* and *PIK3CA* mutations, and 1 presented co-occurring *EGFR* and *KRAS* mutations. In the non-*EGFR*-mutated subgroup, we detected 21 patients with single *KRAS* mutations, 1 with a single *BRAF* mutation, 1 with a *BRAF*/*PIK3CA* co-mutation, 1 with a *BRAF*/*KRAS* co-mutation, and 1 with a *KRAS*/*PIK3CA* co-mutation. Interestingly, only a single patient in the entire cohort harbored an isolated *PIK3CA* mutation. In all other cases, *PIK3CA* alterations were detected either at diagnosis (*N* = 5) or at progression (*N* = 4) alongside another primary driver mutation. Furthermore, these co-occurring *PIK3CA* mutations generally presented with lower VAFs compared to the primary driver (Supplementary Table S1), suggesting their role as subclonal populations potentially involved in primary or acquired resistance mechanisms.

## 4. Discussion

### 4.1. Performance Comparison Between Sysmex NGS and cobas

In this study, we compared the performance of the NGS-based Plasma-SeqSensei™ Solid Cancer IVD assay (Sysmex) to the FDA-approved real-time PCR cobas^®^ EGFR Mutation Test v2 (Roche) in a cohort of 129 patients with advanced NSCLC. Our results demonstrated an excellent overall agreement between the two methodologies, with an Overall Percent Agreement (OPA) of 96.1% and a Cohen’s Kappa of 0.89, indicating almost perfect concordance. These findings are consistent with previous reports in the literature. For instance, comparisons between targeted NGS panels (e.g., the Oncomine Dx Target Test) and the cobas assay in non-squamous NSCLC tissue samples have essentially confirmed the equivalence of the two methods, with minor discrepancies mostly attributable to their inherently different analytical natures [42]. Similarly, an overall concordance of 76.1% (Cohen’s Kappa = 0.59) between the TruSight Tumor 15 NGS assay and cobas in tissue biopsies has previously been reported, alongside a noted high rate of false-positive *EGFR* exon 20 insertions using quantitative PCR [43]. We did not observe any false positives in exon 20 within our cohort; however, this discrepancy is likely explained by the fact that FFPE processing often introduces artifacts in tissue samples, whereas our study exclusively utilized liquid biopsies. Furthermore, longitudinal cfDNA testing has highlighted platform-dependent variability: while certain assays (such as the Oncomine™ Lung cfDNA assay) showed some non-concordance with cobas, other targeted NGS panels demonstrated near-perfect concordance [44]. More recently, the Sysmex Plasma-SeqSensei™ Solid Cancer Kit was evaluated against cobas in a cohort of 31 liquid biopsies, yielding perfect concordance for *EGFR* mutations (Kappa = 0.86; 95% CI: 0.67–1.00) [45]. Collectively, these results, albeit frequently derived from small cohorts, highlight that NGS offers analytical performance comparable to that of standard qPCR for *EGFR* mutation detection. The high sensitivity and specificity of the Sysmex kit are strongly corroborated by broader validations across large cohorts of advanced NSCLC plasma samples (*N* = 212) [41]. Comprehensive comparisons with other routine targeted assays, such as the OncoBEAM^TM^ EGFR V2 and a custom NGS panel, have confirmed the high analytical sensitivity and clinical reliability of this platform across varying cfDNA inputs, highlighting the capability of the Sysmex assay in detecting actionable mutations, perfectly aligning with our observations. Nevertheless, the technological heterogeneity of NGS platforms must be carefully considered, necessitating rigorous clinical validation for each specific panel.

The quantitative relationship between the two assays was also evaluated. A strong, non-linear correlation was observed between the ISQ provided by cobas and the VAF quantified by Sysmex (Spearman’s *ρ* = 0.61; *p* < 0.001) for *EGFR* mutations, aligning with the existing literature [45,46,47]. Notably, a quadratic regression model better described this relationship, demonstrating that VAF increased significantly at higher ISQ values (β = 0.13, *p* = 0.001). However, when isolating patients with ISQ values < 10, the quantitative correlation was lost, and the median VAF dropped significantly (19.0% for ISQ > 10 vs. 2.0% for ISQ < 10, *p* = 0.003). This loss of correlation in the gray zone of low-abundance mutations is likely driven by stochastic variation inherent to PCR amplification near the assay’s limit of detection (LOD). Consequently, this finding emphasizes that, while both methods are highly reliable for qualitative detection, VAF and ISQ values cannot be reliably compared across platforms for longitudinal surveillance or minimal residual disease (MRD) monitoring.

Notably, the Sysmex platform provides absolute quantification of mutant molecules for each detected alteration. This quantitative feature intrinsically supports the longitudinal monitoring of tumor dynamics, provided the same analytical platform is consistently employed, although dynamic tracking was not directly investigated in the present study. Consequently, this targeted NGS panel effectively bridges the gap between single-gene testing and comprehensive genomic profiling (CGP). It allows for the detection of ESCAT-level actionable mutations at a relatively low cost while maintaining exceptional sensitivity, thereby potentially facilitating MRD monitoring. Although the covered gene list is highly focused, it accurately reflects the essential molecular targets required in routine clinical practice. This ensures their reliable detection even at extremely low VAFs, ultimately guaranteeing patient access to appropriate targeted therapies. Nevertheless, the implementation of NGS inherently carries certain limitations, including the necessity of batching 8 to 16 samples per run, a longer hands-on time, and higher overall costs compared to standard real-time PCR, alongside the requirement for a fully equipped molecular laboratory. On the other hand, a major advantage of this assay is its fully automated, on-site bioinformatics workflow. This feature effectively eliminates the need for specialized bioinformatics personnel or complex informatics infrastructure (e.g., dedicated servers), while remaining strictly compliant with IVD requirements.

### 4.2. Enhanced Sensitivity and Rare Variant Detection via Sysmex NGS

Despite the high overall concordance, our analysis underscored the distinct advantages and broader genomic coverage provided by the NGS approach. Sysmex successfully identified *EGFR* mutations in 4 patients (3.1%) who were initially classified as wild-type by the cobas test. Notably, one of these discordant cases (Sample #22) harbored both an exon 19 deletion and a p.(G724S) mutation (VAF, 0.45%), a rare variant located in exon 19 known to potentially reduce the clinical efficacy of osimertinib [48]. Because the cobas^®^ v2 is a targeted assay strictly designed to amplify predefined mutations, it inherently fails to identify rare or novel variants, such as p.G724S, which are captured by the broader interrogation window of amplicon-based NGS. The remaining three discordant cases (samples #1, #117, and #120) rescued by Sysmex presented with classic sensitizing *EGFR* mutations, p.(L861Q), and p.(L858R), but at critically low allelic frequencies (VAF < 0.5%). Furthermore, within the concordant cohort, Sysmex identified concurrent *EGFR* resistance mechanisms in two patients (Sample #23, #63) that were entirely missed by cobas. Specifically, the detection of p.(T790M) and p.(C797S) mutations proved instrumental in understanding these patients’ progression on prior EGFR-TKI therapies [49]. This underscores the ability of the Sysmex NGS technology to achieve robust detection even at VAF levels that fall below the effective LOD of standard real-time PCR assays used in clinical practice. The single false-negative case by Sysmex (an exon 19 deletion detected by cobas) was likely caused by extreme allele sparsity (ISQ = 8.9). While traces of the mutation were visible in the raw BAM sequencing files, the VAF was orders of magnitude below the software’s hard-coded filtering threshold. At such ultra-low concentrations, the physical distribution of mutant DNA fragments in plasma can become stochastic. It is highly probable that the aliquot of plasma used for the real-time PCR assay contained the few available mutant fragments, whereas the aliquot used for NGS library preparation contained zero or insufficient copies to overcome the bioinformatic noise threshold. Furthermore, from a technical perspective, amplicon-based NGS methods can occasionally suffer from allele dropout when amplifying large indels (such as Exon 19 deletions) at near-LOD limits, whereas targeted real-time PCR probes might retain marginal superiority in specifically capturing these known structural breakpoints. To definitively resolve this discrepancy, droplet digital PCR confirmation would be required to determine whether this represents a true platform discordance or a consequence of both methods operating at the stochastic edge of detection, provided sufficient plasma is still available.

Crucially, our logistic regression analysis revealed that the Sysmex assay achieved its highest detection rate when the cfDNA input exceeds 20 ng (OR = 4.41 for inputs ≥ 20 ng, *p* = 0.006) (Supplementary Table S3). This highlights that at lower inputs, the assay’s detection capabilities are limited by both technical constraints and biological factors. The official LOD for the Sysmex kit is established at a VAF of 0.07% (or 7 mutant molecules). However, this optimal performance is only guaranteed with an input of 43 ng (approximately 13,000 genome equivalents). While the manufacturer claims the assay can operate with inputs as low as 5.7 ng, our data show that overall sensitivity is progressively impaired. In our routine setting, 20 ng emerged as the critical lower boundary to ensure diagnostic reliability, aligned with the current literature [50]. At this 20 ng threshold (roughly 6000 genome equivalents), a 0.07% VAF equates to merely 4.2 mutant molecules, which falls below the kit’s absolute cut-off of 7 molecules. Consequently, the actual theoretical sensitivity limit at a 20 ng input mathematically adjusts to 0.14%. The 20 ng threshold should be interpreted as a practical safety margin rather than a strict analytical minimum. cfDNA inputs below this level likely indicate low tumor shedding, presenting a biological constraint that inherently compromises the overall sensitivity of the assay.

### 4.3. Clinical Value of Co-Mutation Profiling by NGS

**Figure 3:**
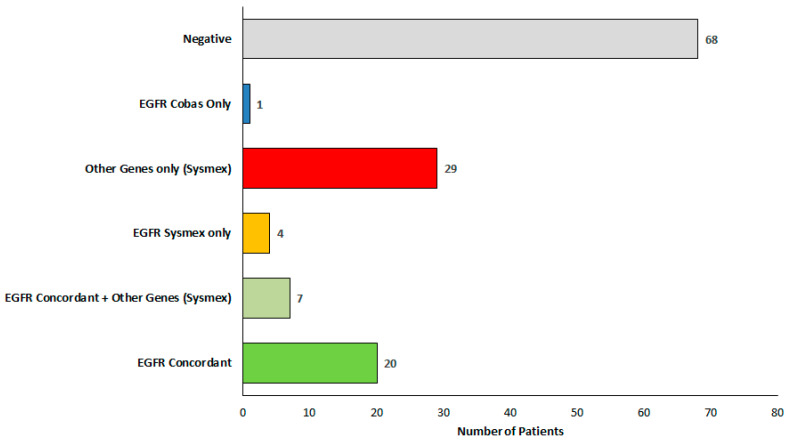
Schematic representation of the Sysmex NGS advantage in the detection of *EGFR* and co-occurring mutations.

Perhaps the most significant clinical advantage of the NGS approach observed in our cohort was its ability to reveal a comprehensive molecular landscape beyond isolated *EGFR* alterations (Figure 3). While the cobas workflow is confined to a single gene, the Sysmex panel identified potentially actionable or prognostically relevant co-mutations in 7 *EGFR*-mutated cases. Moreover, it successfully detected mutations in *KRAS*, *BRAF*, and *PIK3CA* across 29 patients who would have otherwise been classified as negative (wild-type) by the standard single-gene PCR workflow.

Overall, *PIK3CA* mutations were identified in 9 patients (either as isolated events or co-occurring with other mutations). These alterations are potentially targetable by novel isoform-selective PI3K inhibitors (e.g., inavolisib and alpelisib) [26,51]; however, it is important to note that these agents are currently under investigation and are not yet formally approved for NSCLC. We identified 7 patients harboring concomitant *EGFR* mutations and secondary genetic alterations, including 6 specific cases of *EGFR*/*PIK3CA* co-mutation. The presence of concurrent *PIK3CA* mutations is a well-documented negative prognostic factor and represents a recognized mechanism of intrinsic resistance to EGFR-TKIs via bypass activation of the PI3K/AKT/mTOR pathway [23,24]. Given the prevalent sub-clonal nature of *PIK3CA* alterations, combination therapy including an EGFR-TKI and these novel isoform-selective PIK3CA inhibitors should be considered in future clinical trials [26]. Some ongoing early-phase trials and basket studies are actively investigating the use of these novel PIK3CA inhibitors with osimertinib (ClinicalTrials.gov identifier: NCT05284994). Regarding other actionable genes, 5 out of the 24 *KRAS*-mutated patients harbored the specific p.(G12C) mutation, rendering them eligible for targeted therapies such as sotorasib [52] and adagrasib [53]. *BRAF* mutations were detected in 6 patients, including 2 cases of the *BRAF* p.(V600E) variant, which is actionable using approved combination regimens like Dabrafenib plus Trametinib [54] or Encorafenib plus Binimetinib [55]. The routine detection of these complex, multi-gene molecular profiles provides thoracic oncologists with critical biological insights. These high-resolution genomic data allow for more accurate patient stratification, potentially guiding therapeutic decisions toward customized combination strategies or indicating the need for closer clinical monitoring, a level of personalized medicine that conventional single-gene PCR testing simply cannot provide.

## 5. Conclusions

In conclusion, our study demonstrates high analytical concordance between the Sysmex NGS Plasma-SeqSensei™ Solid Cancer IVD kit and the standard cobas^®^ EGFR Mutation Test v2 for *EGFR* profiling in liquid biopsy, with some clinically relevant *EGFR*-mutated patients rescued by the NGS approach. Ultimately, transitioning from single-gene PCR workflows to highly sensitive, multi-gene NGS panels in liquid biopsy is essential to fully realize the potential of precision oncology, ensuring that no actionable therapeutic target is overlooked.

## Figures and Tables

**Table 1:** Characteristics of input cfDNA, ISQ (cobas) and VAF (Sysmex).

Characteristic	*N*	Median (Q1, Q3)
mL plasma	129	4.50 (4.30, 4.80)
cfDNA concentration (ng/mL)	129	12.5 (8.3, 29.5)
Sysmex input qubit (ng)	129	34 * (26, 39)
Genome Equivalent (GE)	128	7203 (5696, 8255)
Sysmex input (ng) from GE	128	31 * (24, 35)
ISQ (cobas)	28	10.4 (6.6, 15.7)
VAF all mutations (Sysmex)	74	1.7 (0.5, 11.2)
VAF *EGFR* (Sysmex)	35	1.7 (0.5, 11.2)

GE = Genome Equivalents; VAF = Variant Allele Fraction; ISQ = Semi-Quantitative Index; * ≥20 ng recommended.

**Table 2:** Concordance of *EGFR* mutation detection between cobas and Sysmex NGS assays (*N* = 129).

	Sysmex *EGFR* Positive	Sysmex *EGFR* Negative	Total
cobas *EGFR* Positive	27	1	28 (21.7%)
cobas *EGFR* Negative	4	97	101 (78.3%)
Total	31 (24.0%)	98 (76.0%)	129
Concordance Metrics			
Overall Agreement	96.1% (124/129)		
Cohen’s Kappa (95% CI)	0.89 (0.80–0.98)		
McNemar’s Test *p*-value	0.371		
Comparative Metrics			
Detection Rate Difference (Sysmex vs. cobas)	2.3% (95% CI: −8.7–13.3)		
Proportion Comparison *p*-value (Chi-squared)	0.767		

CI = confidence intervals.

**Table 3:** (**A**) Discordant cases. (**B**) Partially concordant cases.

(**A**)		
**Sample ID**	**Results of cobas^®^ EGFR Mutation Test v2 (ISQ)**	**Results of Plasma-SeqSensei™ Solid Cancer IVD Kit (VAF)**
1	Not detecte d	p.(L861Q) 0.22%
		p.(T790M) 0.11%
22	Not detected	p.(E746_S752delinsV) 0.83%
	Not evaluable	p.(G724S) 0.45%
91	DEL19 (8.9)	Not detected
117	Not detected	p.(L858R) 0.46%
120	Not detected	p.(L861Q) 0.14%
(**B**)		
**Sample ID**	**Results of cobas^®^ EGFR Mutation Test v2 (ISQ)**	**Results of Plasma-SeqSensei™ Solid Cancer IVD Kit (VAF)**
23	DEL19 (8.99)	p.(L747_A750delinsP) 0.69%
	Not detected	p.(T790M) 0.35%
63	p.(L858R) (9.36)	p.(L858R) 4.56%
	Not evaluable	p.(C797S) 1.67%

## Data Availability

The raw aligned sequencing data (BAM files) generated in this study are openly available in the NCBI Sequence Read Archive (SRA) under the BioProject accession number PRJNA1451442 (https://www.ncbi.nlm.nih.gov/sra/PRJNA1451442 accessed on 27 July 2026).

## Supplementary Materials

The following supporting information can be downloaded at https://www.mdpi.com/article/10.3390/diagnostics16152392/s1, Table S1: Molecular Database of the whole population; Table S2: Quantitative relationship between cobas ISQ and Sysmex NGS VAF; Table S3: Univariate logistic regression analysis of factors associated with successful gene mutation detection using Sysmex NGS.

## Abbreviations

The following abbreviations are used in this manuscript:

aNSCLC	advanced Non-Small-Cell Lung Cancer
cfDNA	circulating cell-free DNA
CI	Confidence Interval
ctDNA	circulating tumor DNA
dsDNA	double-stranded DNA
ESCAT	ESMO Scale for Clinical Actionability of molecular Targets
FDA	Food and Drug Administration
FFPE	Formalin-Fixed Paraffin-Embedded
GE	Genome Equivalents
HS	High Sensitivity
IQR	Interquartile Range
ISQ	Semi-Quantitative Index
IVD	In Vitro Diagnostic
LOD	Limit of Detection
LV	Large Volume
MGAC	Magnesium Acetate
MMX	Master Mix
MRD	Minimal Residual Disease
NGS	Next-Generation Sequencing
NSCLC	Non-Small-Cell Lung Cancer
OPA	Overall Percent Agreement
OR	Odds Ratio
PCR	Polymerase Chain Reaction
PMPF	Post-Market Performance Follow-up
qPCR	quantitative Polymerase Chain Reaction
RT-PCR	Real Time Polymerase Chain Reaction
TKI	Tyrosine Kinase Inhibitors
VAF	Variant Allele Fraction
