# Supplementary material for: Comparing Real-Time PCR and Amplicon-Based NGS for Routine EGFR Liquid Biopsy Profiling
Source: Diagnostics (Basel). 2026 Jul 30;16(15):2392. doi: 10.3390/diagnostics16152392 (PMC13464861; doi:10.3390/diagnostics16152392)
Supplement: Supplementary file 1 [file diagnostics-16-02392-s001.zip › Supplementary tables.pdf]

Supplementary Table S2. Quantitative relationship between Cobas ISQ and Sysmex NGS VAF.

Analysis Type	Variables / Categories	*N*	Statistical Estimate / Median (IQR)	p-value
Correlation Models				
Spearman's Rank	ISQ vs. VAF		ρ = 0.61	< 0.001*
Quadratic Regression	ISQ^2 vs. VAF		β = 0.13 (95% CI: 0.08 – 0.18)	< 0.001*
Stratified Comparison				
Wilcoxon Rank Sum Test	ISQ < 10 (Reference)	14	VAF: 2.0% (1.0 – 3.0)	-
	ISQ ≥ 10	14	VAF: 19.0% (9.0 – 46.0)	0.003*

*p < 0.05 (statistically significant)

Supplementary Table S3. Univariate logistic regression analysis of factors associated with successful gene mutation detection using Sysmex NGS.

Variable	Category / Measure	Total	Mutation not detected, *N* (%)	Mutation Detected, *N* (%)	Odds Ratio (95% CI)	p-value
input qubit	continuous	129			1.05 (1.015, 1.097)	0.008*
input qubit	>=43	13	6 (46%)	7 (54%)	reference	
	30-43	69	32 (46%)	37 (54%)	0.99 (0.29, 3.28)	>0.9
	20-30	28	15 (54%)	13 (46%)	0.74 (0.19, 2.79)	0.7
	5.7-20	19	16 (84%)	3 (16%)	0.16 (0.03, 0.78)	0.03*
Input from GE	continuous	128			1.08 (1.03 - 1.12)	<0.001*
Input from GE	>=43	11	2 (18%)	9 (82%)	reference	
	30-43	59	27 (46%)	32 (54%)	0.26 (0.02, 0.67)	0.11
	20-30	33	20 (61%)	13 (39%)	0.14 (0.02, 0.67)	0.024*
	5.7-20	25	20 (80%)	5 (20%)	0.06 (0.01 - 0.29)	0.002*
Input Threshold	< 20 ng	25	20 (80%)	5 (20%)	reference	-
	≥ 20 ng	103	49 (48%)	54 (52%)	4.41 (1.64 - 14.1)	0.006 *
Clinical Status	Diagnosis	88	44 (50%)	44 (50%)	reference	-
	Progression	40	24 (60%)	16 (40%)	0.67 (0.31 - 1.41)	0.3

GE = Genome Equivalents; *p < 0.05 (statistically significant)
